# Electrocardiogram Characteristics and Prognostic Value in Light-Chain Amyloidosis: A Comparison With Cardiac Magnetic Resonance Imaging

**DOI:** 10.3389/fcvm.2021.751422

**Published:** 2021-12-06

**Authors:** Xinli Guo, Zhian Chen, Ke Wan, Rizhen Song, Tingjie Yang, Yuanwei Xu, Qing Zhang, Kevin Michael Alexander, Yuchi Han, Yucheng Chen

**Affiliations:** ^1^Department of Cardiology, West China Hospital, Sichuan University, Chengdu, China; ^2^State Key Laboratory of Biotherapy and Cancer Center, West China Hospital, Sichuan University, Chengdu, China; ^3^Department of Geriatrics and National Clinical Research Centre for Geriatrics, West China Hospital, Sichuan University, Chengdu, China; ^4^Stanford Amyloid Center, Division of Cardiovascular Medicine, Stanford University School of Medicine, Stanford, CA, United States; ^5^Stanford Cardiovascular Institute, Stanford University School of Medicine, Stanford, CA, United States; ^6^Department of Medicine (Cardiovascular Division), University of Pennsylvania, Philadelphia, PA, United States; ^7^Center of Rare Diseases, West China Hospital, Sichuan University, Chengdu, China

**Keywords:** light-chain amyloidosis, cardiac involvement, electrocardiogram, cardiac magnetic resonance, prognosis

## Abstract

**Background:** An electrocardiogram (ECG) is a simple and cheap non-invasive tool that shows various abnormalities and has prognostic value for patients with light-chain amyloidosis (AL). The present study aimed to explore the association between ECG characteristics and cardiac magnetic resonance (CMR)-detected amyloid burden and to investigate the prognostic value of ECG in AL amyloidosis.

**Methods:** We prospectively enrolled 99 patients with AL amyloidosis (56 male patients; median age, 58 y). Detailed clinical information, 12-lead ECG, and CMR data were collected. All patients were followed up longitudinally, and the endpoint was all-cause mortality. ECG characteristics were analyzed and correlated with the degree of late gadolinium enhancement (LGE) and extracellular volume (ECV) by T1 mapping on CMR. The prognostic value of ECG characteristics was analyzed using Kaplan–Meier survival analysis and multivariate Cox regression.

**Results:** During a median follow-up period of 33 months, 69 of the 99 patients died. Fragmented Q wave-R wave-S wave (QRS), pathological Q waves, the Sokolow index, QRS duration, and voltages were significantly associated with the extent of LGE, native T1, and ECV by CMR (*p* < 0.05). Fragmented QRS and Sokolow index showed independent prognostic value in AL amyloidosis (*p* = 0.001; *p* = 0.026, respectively). Fragmented QRS remained independent after adjusting for clinical values (hazard ratio: 2.034; 95% confidence interval: 1.148–3.603; *p* = 0.015). However, no ECG characteristics were independent predictors for prognosis in AL amyloidosis when LGE and ECV were included in the multivariate analysis.

**Conclusion:** ECG abnormalities showed significant association with CMR indicators of amyloid burden. Fragmented QRS has an independent prognostic value in AL amyloidosis and could be used as an alternative marker when CMR is not available.

## Introduction

Amyloidosis is a rare systemic disease caused by tissue infiltration of misfolded, insoluble proteins, which leads to multiorgan dysfunction (1–4). Cardiac amyloidosis occurs secondary to the deposition of protein fibrils in cardiac tissue, which gradually disrupts cardiac structure and function, leading to progressive myocardial dysfunction, arrhythmia, and heart failure (2, 5). Previous data demonstrated that cardiac involvement occurred in as many as half of all patients with light chain amyloidosis (AL) (6). Recent studies have proposed that a greater cardiac amyloid burden was associated with increased disease severity and worse prognosis (7–10). Therefore, it is important to evaluate the extent of cardiac involvement in patients with AL amyloidosis and predict outcomes and guide treatment selection.

Electrocardiography, as a simple and cheap non-invasive tool, reveals various abnormalities in patients with AL cardiac amyloidosis. Amyloid infiltration in the myocardium can lead to bradycardia, conduction abnormalities, atrial fibrillation, and even sudden cardiac death (11, 12). Other ECG signs that might be present include low-voltage complexes, the pseudo infarction pattern in the inferolateral leads, fragmented Q wave-R wave-S wave (QRS), and prolonged intervals (13–15). Patients with cardiac amyloidosis with arrhythmic manifestations might have more advanced disease and a worse prognosis, with the attributed mechanism of sudden cardiac death in most patients being pulseless electrical activity (16). Recently, some ECG characteristics have been used to measure the presence of cardiac involvement and prognosis in patients with AL amyloidosis, such as fragmented QRS (fQRS) and the Skolow index (14, 17, 18). However, the ability of 12-lead ECG abnormalities to identify the extent of cardiac involvement has not yet been studied, and its independent role in prognosis remains unclear.

To date, cardiac magnetic resonance imaging has been considered an excellent non-invasive test to measure cardiac amyloid infiltration. Late gadolinium enhancement (LGE) (19), myocardial native T1, and the extracellular volume (ECV) (7, 8, 20) have been used for tissue characterization. These techniques have emerged as early disease markers to detect cardiac involvement and to estimate prognosis in patients with cardiac amyloidosis. However, the technical complexity, cost, and patient-specific factors limit the widespread use of CMR. Therefore, exploring a simple screening protocol utilizing routine ECG when CMR is not available would be useful.

The present study aimed to identify simple 12-lead ECG characteristics that can stratify disease severity and prognosis in patients with AL amyloidosis.

## Materials and Methods

### Population

We studied prospectively patients who were diagnosed with systemic AL amyloidosis from September 2013 to December 2018 at West China Hospital. We excluded those patients who did not have any one of the following examinations: laboratory tests, ECG, and contrast-enhanced CMR. We also excluded patients with other potential causes of low-voltage QRS (chronic obstructive pulmonary disease, severe peripheral edema, or large pericardial effusion). Furthermore, patients with poor ECG and CMR image qualities were excluded. In total, 99 patients were enrolled into the present study. The diagnosis of AL amyloidosis was confirmed using immunohistochemistry and Congo red staining of a biopsy taken from an involved organ or tissue. Cardiac involvement was defined according to a left ventricle (LV) wall thickness of more than 12 mm with no other potential cardiac cause, as shown by echocardiography or CMR (21). This study was approved by the Institutional Review Board, and written informed consent was obtained from all subjects.

### Electrocardiogram

A standard 12-lead ECG was obtained from each patient at the time of the first clinical evaluation after being referred to our center. We measured the Q wave to T wave (QT) interval, QT corrected (QTc) interval, QRS duration, and QRS complex voltages, and analyzed the tracings for atrial fibrillation, atrial flutter, pathological Q waves, and fQRS waves. The QTc interval was corrected by the QT interval for the heart rate based on the formula (QTc = QT/√RR). Voltages were measured in millimeters (1 mm =0.1 mV) after calibration, and the duration was measured in milliseconds (1 mm =0.04 s). A pseudo infarct pattern was defined as a pathological Q wave in at least two adjacent leads without obstructive coronary artery disease or no prior myocardial infarction history. fQRS was defined as the presence of various RSR' patterns and included a notching or additional R or S wave, or the presence of > 1 R′ in two consecutive leads corresponding to a major coronary artery region (22). The Sokolow index was calculated by the amplitude of the S wave in V1 plus the R wave in V5 or V6, and a value of more than 1.5 mv was considered as a normal Sokolow index (23). The sum of the entire QRS complex voltage, including leads I, II, III, aVF, aVR, and aVL, was considered to be the total limb lead voltage; similarly, the sum of leads V1–V6 was considered to be the total precordial lead voltage (15).

### Cardiovascular Magnetic Resonance (CMR) Protocol and Imaging Analyses

All cardiovascular magnetic resonance imaging was performed using a 3.0-T scanner (Magnetom Tim Trio; Siemens Healthcare, Erlangen, Germany) using a 32-channel phased-array cardiac coil during breath holding. According to a standardized protocol, steady-state free-precession cine images were obtained in continuous short axes and in the long axis (two-, three-, and four-chamber views). The parameters were the following: repetition time ms/echo time ms, 3.4/1.3; flip angle, 50°; field of view, 320–340 mm; matrix, 256 × 144; and slice thickness, 8 mm with no gap. Gadolinium-enhanced images were obtained in the same slices of cine images at 10–15 min after injection with gadopentetate dimeglumine (0.15 mmol per kilogram of body weight, Magnevist; Bayer Schering Pharma, Berlin, Germany). LGE images were acquired using an inversion recovery sequence with phase-sensitive reconstruction. T1 mapping images of three short-axis slices (basal, mid, and apical) were obtained using a modified Look-Locker inversion recovery sequence and a fixed 5 (3) 3/4 (1) 3 (1) 2 scheme. Post-contrast T1 mapping was repeated using the same prescription as the pre-contrast T1 mapping at 15 min after contrast agent injection (repetition time ms/echo time, 2.9/1.12; flip angle, 35°; bandwidth, 930 Hz; TI of first experiment, 100 ms; TI increment, 80 ms; 2 × parallel imaging, matrix size, 192 × 144; in-plane spatial resolution, 2.4 × 1.8 mm; and the total acquisition time was 11 heart beats).

Ventricular volumes, masses, and ejection fractions were assessed from the short-axis images with the QMass v7.6 software (Medis, Leiden, The Netherlands). Papillary muscles were included as part of the ventricular volumes and were excluded from the myocardial mass. Ventricular volumes and LV mass were standardized by the body surface area.

LGE types were divided into three groups using the following criteria: (1) no or non-specific LGE; (2) sub-endocardial LGE; and (3) transmural LGE (24, 25). Native T1 and ECV maps were calculated using the QMap 2.2.6, software (Medis). The epicardial and endocardial boundaries were manually traced on the motion corrected pre- and post-contrast T1-mapping images at the two LV short-axis levels, excluding near wall blood, epicardial fat, and areas representing Gibbs artifacts. Blood T1 was gained by tracing regions of interest (ROIs) in the blood pool in the pre- and post-contrast T1 mapping images. ECV was analyzed by T1 mapping based on the following formula: ECV = (1-hematocrit) ^*^ [(1/T1myo post-1/T1myo pre)/(1/T1 blood post-1/T1 blood pre)]. Mean ECV, myocardial native T1 (T1myo), and post T1 were acquired as the averages of the ECV and myocardial T1 values at two slices, respectively.

### Follow-Up

The endpoint for this study was defined as all-cause mortality. Mortality was assessed by medical records or phone interviews with the patient's family. There was no loss of follow-up in the entire cohort. The patients were followed up until 6 August 2019 or censored if they were alive.

### Statistical Analyses

Data were expressed as the mean ± standard deviation (SD) for continuous variables and number and percentage (*n*, %) for categorical variables. Differences between groups were tested using Student's *t*-test or one-way ANOVA for continuous variables and using Fisher's exact test or the χ^2^ test for categorical variables, as appropriate. The statistical comparison between groups was performed using scatter plots and histograms to describe the trend of variables. Kaplan–Meier curves and multivariate Cox proportional hazard regressions were used for the survival analyses. All variables with a *p* < 0.05 in the univariate Cox regression were included in the stepwise multivariate Cox regression analysis. All tests were two-sided, and *p* < 0.05 was considered to be statistically significant. SPSS statistical software (version 21.0; IBM Corp., Armonk, NY, USA) and GraphPad Prism (version 7.0; GraphPad Inc., La Jolla, CA, USA) were used for all statistical analyses.

## Results

### Demographic and Baseline Clinical Data

**Figure 1:**
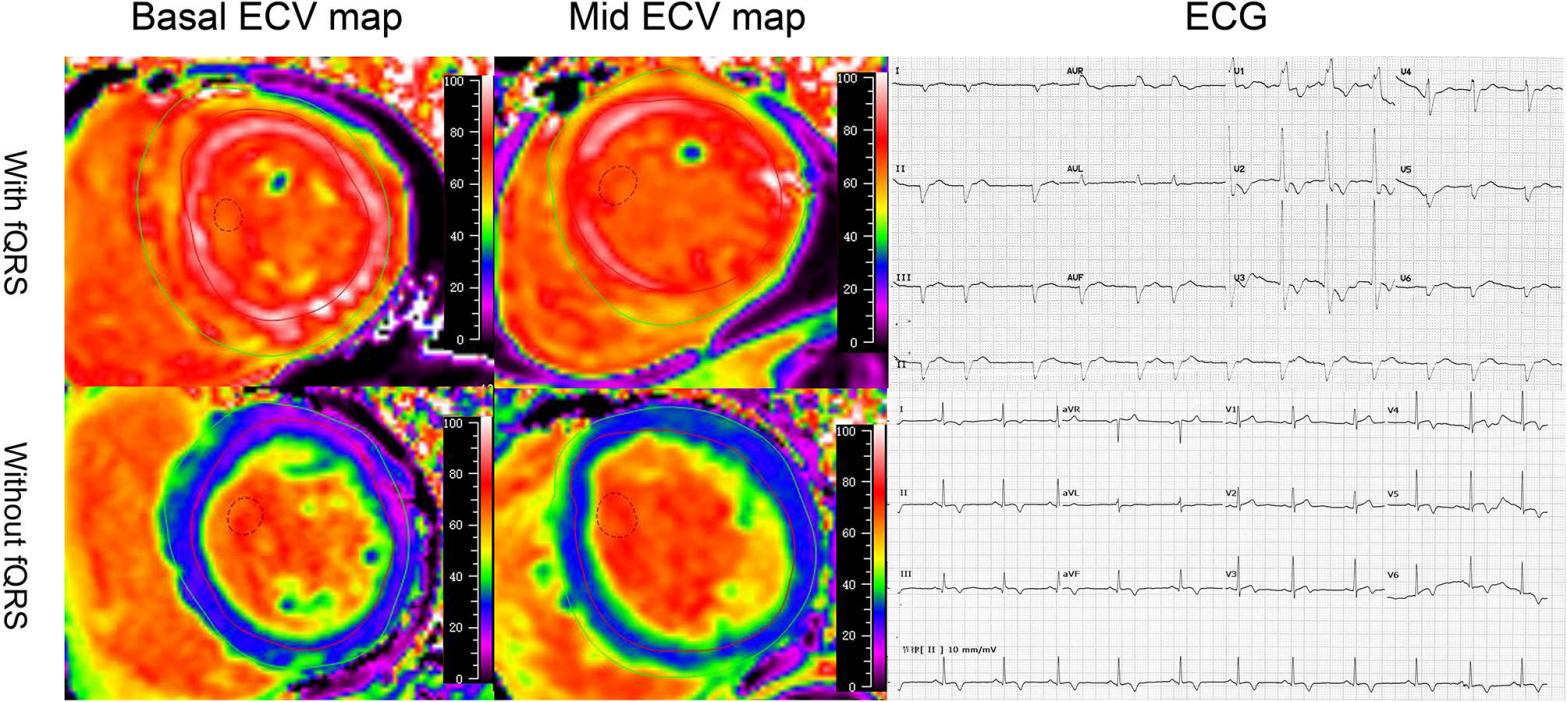
Illustrative case examples of the extracellular volume and electrocardiogram in patients with AL amyloidosis. The patients who presented with fragmented QRS on ECG showed high-basal and mid-ECV value compared with the patients who were absent with fragmented QRS.

Of the 99 patients (male, 56 patients; median age, 58 years) included in this study, 69 patients died, and a total of 88 patients were diagnosed with cardiac involvement. Apart from a low Sokolow index (73.7%), we found that the common ECG abnormalities were fQRS (63.3%) (Figure 1), followed by low limb lead voltages (67.7%), pathological Q waves (61.6%), a prolonged QTc interval (51.5%), low precordial lead voltages (35.4%), and prolonged QRS (9.1%). The median follow-up time for this group was 33 months [interquartile range (IQR) 32–39 months]. In terms of the disease stage, 5 (5%), 22 (22%), and 72 (73%) of the patients with AL amyloidosis had mayo Stages I, II, and III disease, respectively. Compared with the patients who survived, the patients who died had worse baseline cardiac function [New York Heart Association (NYHA) classes III–IV: 86.3 vs. 13.7%, *p* < 0.001; mean N-terminal pro-B-type natriuretic peptide (NT-ProBNP): 9,350 pg/ml vs. 4,054 pg/ml, *p* < 0.001] and were more likely to have fQRS (82.5 vs. 17.5%, *p* < 0.001) (Table 1). In addition, longer QRS duration and a lower Sokolow index were observed significantly more frequently in patients who died compared with those in the patients that survived (*p* = 0.037, *p* = 0.044, respectively). There were no significant differences in age, sex, body surface area (BSA), heart rate, myocardial markers, creatinine, estimated glomerular filtration rate (eGFR), serum free light chains, hematocrit, and other ECG parameters, such as the QTc interval and the total limb and precordial lead voltages. Other patient clinical characteristics and CMR data are reported in Table 1, respectively.

**Table 1:** Comparison of baseline clinical characteristics of patients with or without all-cause mortality.

**Characteristics**	**All-cause mortality (-) (*n* = 30)**	**All-cause mortality (+) (*n* = 69)**	***p***
**Demographic characteristics**			
Age (years)	57 ± 11	59 ± 11	0.659
BSA (kg/m^2^)	1.6 ± 0.2	1.6 ± 0.1	0.114
NYHA class III-IV (%)	7 (13.7)	44 (86.3)	**<0.001**
HR (beats/min)	84 ± 13	84 ± 16	0.080
SBP (mmHg)	118 ± 21	109 ± 15	**0.029**
DBP (mmHg)	76 ± 14	70 ± 12	0.077
**Laboratory tests**			
NT-ProBNP (pg/mL)	4,054 ± 5,547	11,542 ± 10,829	**<0.001**
TnT (ng/L)	148 ± 320	163 ± 152	0.695
Mayo stage 3	16 (22.2)	56 (77.8)	**0.012**
Creatinine (g/dL)	141 ± 227	111 ± 81	0.513
eGFR (mL/min/1.73 m^2^)	85 ± 40	67 ± 31	0.555
FLC-λ (mg/L)	263 ± 1,093	62 ± 136	0.087
FLC-κ (mg/L)	327 ± 1,475	21 ± 87	0.128
HCT (%)	37 ± 6	37 ± 6	0.644
**ECG parameters**			
Fragmented QRS (%)	11 (17.5)	52 (82.5)	**<0.001**
Pathological Q waves (%)	15 (24.6)	46 (75.4)	0.177
AF/Atrial flutter (%)	5 (20.8)	19 (79.2)	0.313
QTc interval (msec)	434 ± 51	448 ± 47	0.142
QRS duration (msec)	88 ± 14	100 ± 24	**0.037**
Total limb voltages (mV)	3.1 ± 1.3	2.5 ± 1.5	0.114
Total precordial voltages (mV)	7.0 ± 2.7	7.8 ± 3.2	0.548
Sokolow index (mV)	1.4 ± 0.7	1.1 ± 0.7	**0.044**
**CMR values**			
Mean native T1 (ms)	1,330 ± 67	1,392 ± 85	**0.001**
Mean post T1 (ms)	479 ± 106	417 ± 98	**0.020**
Mean ECV (%)	42 ± 9	54 ± 11	**<0.001**
Transmural LGE	5 (10.4)	43 (89.6)	**<0.001**
Wall thickness (mm)	11 ± 3	13 ± 3	**0.001**
LVEF (%)	57 ± 11	41 ± 13	**<0.001**
LVESVi (mL/m^2^)	30 ± 12	44 ± 15	**0.006**
LVEDVi (mL/m^2^)	70 ± 18	74 ± 19	0.395
LVMassi (g/ m^2^)	77 ± 24	113 ± 62	**0.018**
RVEF (%)	56 ± 14	40 ± 14	**<0.001**
RVESVi (mL/m^2^)	27 ± 11	45 ± 18	**<0.001**
RVEDVi (mL/m^2^)	63 ± 19	73 ± 25	0.208

*Values are mean ± standard deviation or n (%). The bold values mean P>0.05*.

*BSA, body surface area; NYHA, New York Heart Association; HR, heart rate; SBP, systolic blood pressure; DBP, diastolic blood pressure; NT-ProBNP, N-terminal pro-B-type natriuretic peptide; TnT, troponin-T; eGFR, estimated Glomerular Filtration Rate; FLC, free light chain; HCT, hematocrit; AF, atrial fibrillation; QTc interval, QT corrected interval; ECV, extracellular volume fraction; LGE, late gadolinium enhancement; LV, left ventricular; RV, right ventricular; EF, ejection fraction; ESVi, end systolic volume index; EDVi, end diastolic volume index*.

### Association Between ECG Characteristics and Cardiac Amyloid Burden Evaluated by CMR

#### ECG Values and LGE Patterns

**Figure 2:**
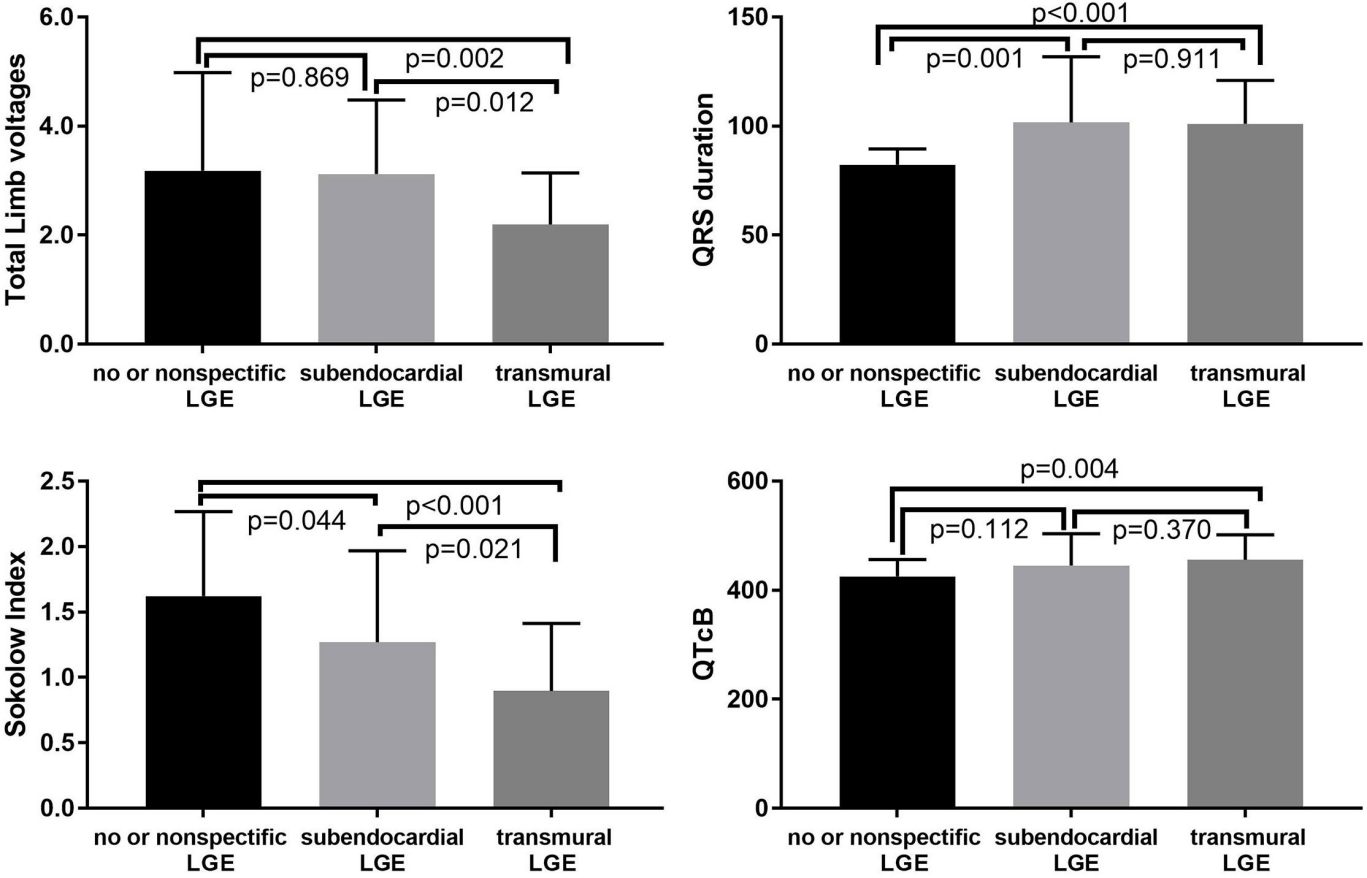
Relationships between the LGE pattern and Sokolow index, QTcB, QRS duration, and total limb voltages.

In the present study, 31 (31.3%), 20 (20.2%), and 48 (48.5%) patients had no or non-specific, subendocardial, or transmural LGE patterns, respectively. There were significant differences in fQRS, pathological Q waves, QTc interval, QRS duration, total limb lead voltages, or the Sokolow index among the three LGE groups (*p* < 0.01) (Table 2). More severe LGE patterns were associated with a lower QRS voltage and longer QRS duration (Figure 2). For the QTc interval, a significant difference was observed between the no or non-specific LGE and transmural LGE groups (*p* = 0.004). However, there was no significant difference among the three groups with regard to atrial fibrillation, atrial flutter, and or total precordial lead voltages.

**Table 2:** ECG parameters among the three patterns of late gadolinium enhancement (LGE).

**Characteristics**	**No or non-specific LGE (31)**	**Subendocardial LGE (20)**	**Transmural LGE (48)**	*p* ^*^
Fragmented QRS (%)	11 (17.5)	12 (19.0)	40 (65.3)^§^, ^‡^	**<0.001**
Pathological Q waves (%)	10 (16.4)	12 (19.7)^§^	39 (63.9)^§^	**<0.001**
AF/Atrial flutter (%)	8 (33.3)	4 (16.7)	12 (50.0)	0.881
QTc interval (msec)	425 ± 31	445 ± 59	456 ± 46^§^	**0.004**
QRS duration (msec)	82 ± 7	102 ± 30^§^	101 ± 20^§^	**<0.001**
Total limb voltages (mV)	3.2 ± 1.8	3.1 ± 1.4	2.2 ± 0.9^§^, ^‡^	**0.003**
Total precordial voltages (mV)	8.4 ± 3.7	6.9 ± 3.3	7.2 ± 2.2	0.240
Sokolow index (mV)	1.6 ± 0.6	1.3 ± 0.7^§^	0.9 ± 0.6^§^, ^‡^	**<0.001**

* *P-value related to the comparison among the three patterns of LGE. The bold values mean P > 0.05*.

§ *P < 0.05 vs. no or non-specific LGE*.

‡ *P < 0.05 vs. subendocardial LGE*.

*BSA, body surface area; NYHA, New York Heart Association; HR, heart rate; SBP, systolic blood pressure; DBP, diastolic blood pressure; NT-ProBNP, N-terminal pro-B-type natriuretic peptide; TnT, troponin-T; eGFR, estimated Glomerular Filtration Rate; FLC, free light chain; HCT, hematocrit; AF, atrial fibrillation; QTc interval, QT corrected interval; ECV, extracellular volume fraction; LGE, late gadolinium enhancement; LV, left ventricular; RV, right ventricular; EF, ejection fraction; ESVi, end systolic volume index; EDVi, end diastolic volume index*.

#### ECG Characteristics and T1 Mapping

**Figure 3:**
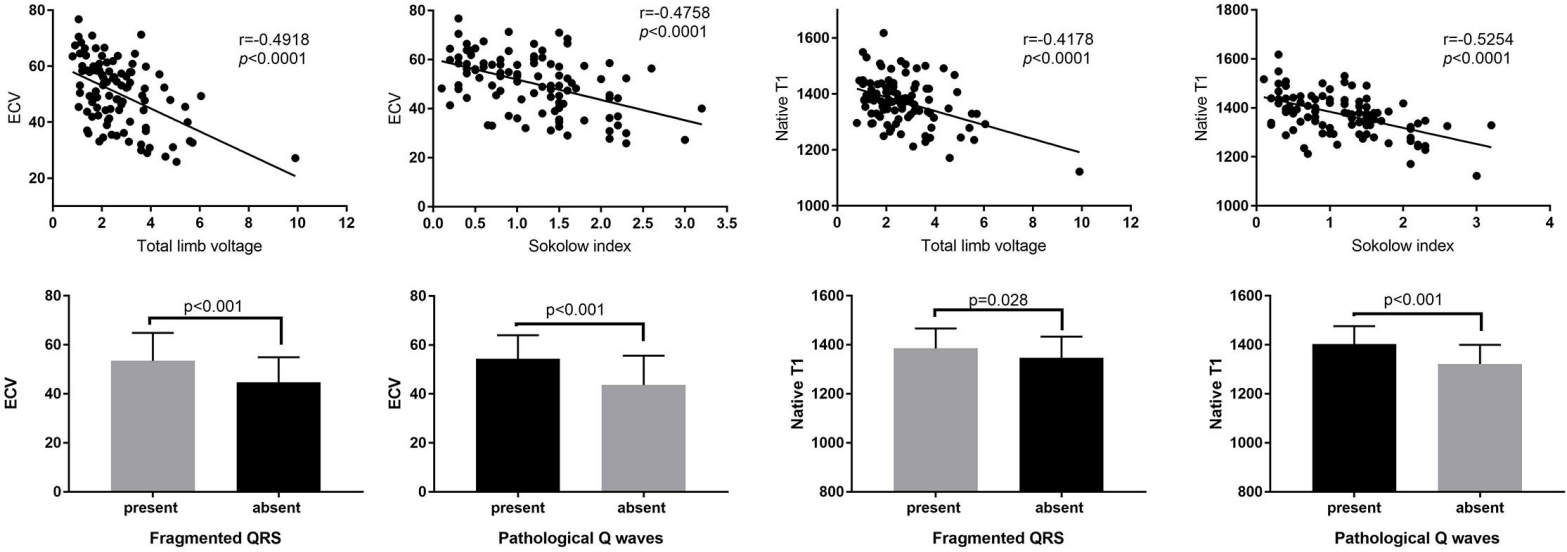
The relationships between ECG parameters and ECV and native T1.

ECG markers were further studied in patients with cardiac amyloidosis and compared with T1 mapping. A scatter diagram illustrated the trend of ECG values and native T1 as well as ECV. An increase in the Sokolow index correlated strongly with a decrease in native T1 and ECV (*r* = −0.5254, *p* < 0.0001; *r* = −0.4758, *p* < 0.0001) (Figure 3). A similar negative correlation was found between total limb lead voltage and the native T1 and ECV (Figure 3). Moreover, the appearance of fQRS and pathological Q waves was associated with higher native T1 and ECV values (*p* < 0.05) (Figure 3).

### Prognostic Value of ECG

**Figure 4:**
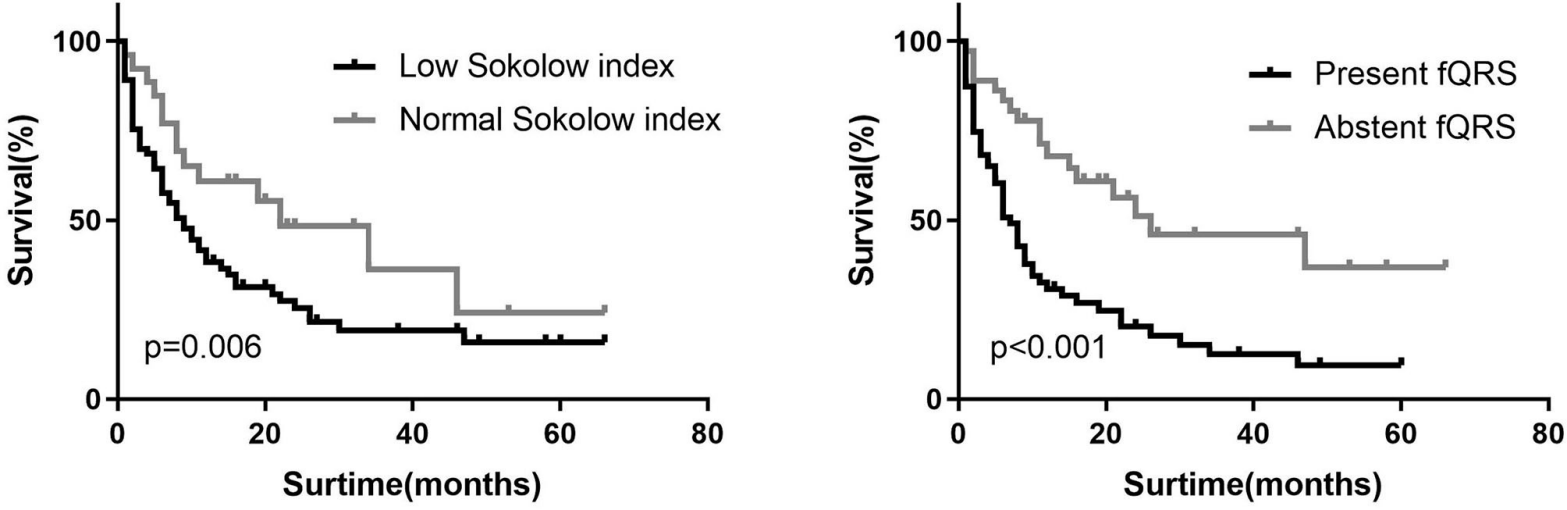
Kaplan–Meier curve for ECG parameters, predicting all-cause mortality in patients with AL amyloidosis.

In the univariate Cox regression analysis of the ECG data, the variables that were associated significantly with mortality were the presence of fQRS or pathological Q waves, QRS duration, the Sokolow index, and total limb lead voltages. In the multivariate analysis (Table 3), only fQRS [χ^2^ = 13.152, hazard ration (HR) 2.471, *P* = 0.001] and Sokolow index (χ^2^ = 7.574; HR, 0.671; *P* = 0.026) were independent predictors. The Kaplan–Meier curves for fQRS and the Sokolow index indicated that the absence of fQRS and a normal Sokolow index were associated with better outcomes among patients with AL amyloidosis (Figure 4). After adjusting for age, sex, and known prognostic predictors, such as NT-ProBNP and troponin-T (TnT) (26), we found that fQRS and NT-ProBNP remained independent predictors (Table 4). When we entered the ECG, clinical, and CMR predictors into the model, however, none of the ECG data remained independently associated with all-cause mortality (Table 4). The final multivariable Cox analysis indicated that the mean ECV [HR, 1.038; 95% confidence interval (CI), 1.012–1.065; *p* = 0.004] and NT-ProBNP [HR, 1.962; 95% (CI), 1.190–3.234; *p* = 0.008] were independent prognostic factors.

**Table 3:** Univariate and multivariate cox proportional hazard regression analysis using ECG values to identify electrocardiographic predictors of all-cause mortality.

		**Univariate analysis**		**Multivariate analysis**	
**Characteristics**	**χ^2^**	**HR (95% CI)**	***p***	**HR (95% CI)**	***p***
Fragmented QRS	13.152	2.673 (1.539–4.641)	**<0.001**	2.471 (1.414–4.318)	**0.001**
Pathological Q waves	4.699	1.736 (1.048–5.877)	**0.032**		
AF/Atrial flutter	0.299	1.160 (0.682–1.972)	0.585		
QTc interval (msec)	1.408	1.003 (0.998–1.007)	0.235		
QRS duration (msec)	6.472	1.012 (1.003–1.021)	**0.012**		
Sokolow index (mV)	7.574	0.603 (0.420–0.866)	**0.006**	0.671 (0.472–0.953)	**0.026**
Total limb voltages (mV)	3.859	0.816 (0.666–0.999)	**0.049**		
Total precordial voltages (mV)	0.390	0.974 (0.897–1.058)	0.532		

*The bold values mean P>0.05. HR, hazard ratio; CI, confidence interval; BSA, body surface area; NYHA, New York Heart Association; HR, heart rate; SBP, systolic blood pressure; DBP, diastolic blood pressure; NT-ProBNP, N-terminal pro-B-type natriuretic peptide; TnT, troponin-T; eGFR, estimated Glomerular Filtration Rate; FLC, free light chain; HCT, hematocrit; AF, atrial fibrillation; QTc interval, QT corrected interval; ECV, extracellular volume fraction; LGE, late gadolinium enhancement; LV, left ventricular; RV, right ventricular; EF, ejection fraction; ESVi, end systolic volume index; EDVi, end diastolic volume index*.

**Table 4:** Cox proportional hazard multivariable models to define the independent impact of ECG characteristics on survival.

	**Univariate analysis**		**Multivariate analysis**	
**Characteristics**	**HR (95% CI)**	***p***	**HR (95% CI)**	***P***
**Clinical model**				
Age	1.002 (0.980–1.026)	0.835		
Male	1.178 (0.728–1.907)	0.504		
NYHA class III-IV	2.515 (1.517–4.169)	**<0.001**		
Lg NT-ProBNP	2.633 (1.843–3.762)	**<0.001**	2.279 (1.440–3.067)	**<0.001**
Lg TnT	1.840 (1.174–2.885)	**0.008**		
Fragmented QRS	2.673 (1.539–4.641)	**<0.001**	2.034 (1.148–3.603)	**0.015**
Sokolow index	0.603 (0.420–0.866)	**0.006**		
**Clinical and CMR model**				
Age	1.002 (0.980–1.026)	0.835		
Male	1.178 (0.728–1.907)	0.504		
NYHA III-IV	2.515 (1.517–4.169)	**<0.001**		
Lg NT-ProBNP	2.633 (1.843–3.762)	**<0.001**	1.962 (1.190–3.234)	**0.008**
Lg TnT	1.840 (1.174–2.885)	**0.008**		
Fragmented QRS	2.673 (1.539–4.641)	**<0.001**		
Sokolow index	0.603 (0.420–0.866)	**0.006**		
Mean native T1	1.005 (1.003–1.007)	**<0.001**		
Mean ECV	1.056 (1.037–1.074)	**<0.001**	1.038 (1.012–1.065)	**0.004**
Transmural LGE	3.444 (2.073–5.723)	**<0.001**		

*The bold values mean P > 0.05. HR, hazard ratio; CI, confidence interval; BSA, body surface area; NYHA, New York Heart Association; HR, heart rate; SBP, systolic blood pressure; DBP, diastolic blood pressure; NT-ProBNP, N-terminal pro-B-type natriuretic peptide; TnT, troponin-T; eGFR, estimated Glomerular Filtration Rate; FLC, free light chain; HCT, hematocrit; AF, atrial fibrillation; QTc interval, QT corrected interval; ECV, extracellular volume fraction; LGE, late gadolinium enhancement; LV, left ventricular; RV, right ventricular; EF, ejection fraction; ESVi, end systolic volume index; EDVi, end diastolic volume index*.

## Discussion

This study investigated the ability of ECG parameters to detect the degree of cardiac involvement in AL amyloidosis and explored the prognostic value of ECG in comparison with CMR. The main study findings were as follows: (1) ECG characteristics in AL amyloidosis were associated significantly with cardiac amyloid burden, as reflected by LGE extent and T1 mapping; (2) both fQRS and a low Sokolow index were significant independent predictors for the survival of patients with AL amyloidosis; (3) fQRS was a significant independent prognostic marker and could be a simple and alternative prognostic factor, especially when CMR is not available.

With recent increasing awareness and improved screening technology, the contemporary estimate of the prevalence of AL amyloidosis is 18–55 cases per 100,000 person-years, leading to ~8% of heart failure hospitalizations within the previous year (27). Cardiac amyloid deposition is a key driver of impaired contractile dysfunction and fatal arrhythmia. CMR has been considered the gold standard imaging technique to assess cardiac amyloid burden and patient prognosis (20). Previous studies have demonstrated that transmural LGE and T1 mapping (native myocardial T1 and ECV) represent advanced cardiac amyloid infiltration and provided incremental information on overall survival (24, 28). In the present study, transmural LGE and ECV values were also found to be associated significantly with outcomes in patients with AL cardiac amyloidosis.

Generally, patients with AL amyloidosis and arrhythmic manifestations might have more advanced disease and worse prognosis (16). Assessing ECG abnormalities in these patients is crucial, although it remains relatively unstudied. A potential mechanism is that the insoluble protein deposits in conduction tissue or within myocardial tissue disrupt normal electromechanical conduction and cause arrhythmias (29). Previous studies have reported that limb lead and Sokolow voltage measurements (17), fQRS (14), and prolonged QTc interval (≥ 483 ms) (30) are associated significantly with all-cause mortality in AL cardiac amyloidosis. A linear correlation between QRS voltages and ECV has been reported recently by Banypersad et al. in a series of 60 patients with AL cardiac amyloidosis (31). In this prospective study, we not only identified a significant association between ECG parameters, such as QRS voltages and the extent of myocardial LGE and ECV, but also that some ECG variables could differentiate transmural LGE from non-transmural myocardial LGE reliably in patients with cardiac amyloidosis. These findings suggested that the ECG parameters could assess and stratify the myocardial amyloid burden and serve as a surrogate when CMR is unavailable. Such a simple, fast, and cheap test might improve the characterization of the extent of myocardial amyloid infiltration and aid in the prognostic assessment of AL cardiac amyloidosis.

Fragmented QRS morphologies are believed to represent conduction block caused by myocardial necrosis or scarring in either ischemic or non-ischemic cardiomyopathy (14, 22, 32). Previous studies found the fQRS increased the risk of adverse cardiac events and was associated with poor prognosis in various heart diseases, including amyloidosis; however, the relationship between fQRS and CMR has not been well-studied (14, 22). Consistent with other studies, our results suggested that fQRS could be used as a marker for the severity of cardiac amyloid involvement and to monitor disease progression in AL cardiac amyloidosis. In particular, fQRS was an independent predictor of survival even when CMR characteristics were not considered. However, compared with CMR, ECG has the advantage of being widely available and easy to analyze. Importantly, we considered that the strong correlation between fQRS and worse cardiac involvement, as detected by CMR in patients with CA, supports previous finding that this index is related to myocardial scarring. Thus, the current study highlights the importance of fQRS for cardiac amyloid detection, risk stratification, and prognosis in patients with AL cardiac amyloidosis.

## Limitations

The present study had several limitations. First, this was a single-center study and included a relatively small population, which is unsurprising, given the rarity of AL amyloidosis. Second, the cardiac amyloid burden was assessed using non-invasive CMR because a minority of patients underwent endomyocardial biopsy. Currently, LGE and T1 mapping are considered powerful tools to assess cardiac amyloid involvement. Third, we excluded some patients whose CMR and ECG imaging were poor, which might have induced bias. Fourth, the ECG parameters that we used were within the limits of commonly evaluated abnormalities on the ECG; however, there might be other potential values measured by ECG that should be explored.

## Conclusion

We demonstrated that 12-lead ECG is a simple tool to stratify myocardial infiltration in patients with AL and might provide useful information when CMR is not available. Fragmented QRS is an independent prognostic marker for a poor outcome, providing important risk stratification of patients with AL amyloidosis.

## Data Availability Statement

The raw data supporting the conclusions of this article will be made available by the authors, without undue reservation.

## Ethics Statement

The studies involving human participants were reviewed and approved by the Institutional Ethics Committee of West China Hospital, Sichuan University. The patients/participants provided their written informed consent to participate in this study. Written informed consent was obtained from the individual(s) for the publication of any potentially identifiable images or data included in this article.

## Author Contributions

XG, ZC, KW, RS, QZ, YH, and YC: guarantors of integrity of entire study. XG, ZC, KW, YH, and YC: literature research. XG, ZC, RS, TY, YX, QZ, and YC: clinical studies. XG, ZC, KW, and YH: statistical analysis. XG, ZC, KW, QZ, YH, KA, and YC: manuscript editing. All authors: study concepts/study design, data acquisition, data analysis/interpretation, manuscript drafting, manuscript revision for important intellectual content, approval of final version of submitted manuscript, and agreed to ensure any questions related to the work were appropriately resolved.

## Funding

This work was supported by a grant from the Project for Disciplines of Excellence, West China Hospital, Sichuan University [Grant No. ZYJC18013].

## Conflict of Interest

The authors declare that the research was conducted in the absence of any commercial or financial relationships that could be construed as a potential conflict of interest.

## Publisher's Note

All claims expressed in this article are solely those of the authors and do not necessarily represent those of their affiliated organizations, or those of the publisher, the editors and the reviewers. Any product that may be evaluated in this article, or claim that may be made by its manufacturer, is not guaranteed or endorsed by the publisher.
